# Use of the Analytic Hierarchy Process for Medication Decision-Making in Type 2 Diabetes

**DOI:** 10.1371/journal.pone.0126625

**Published:** 2015-05-22

**Authors:** Nisa M. Maruthur, Susan M. Joy, James G. Dolan, Hasan M. Shihab, Sonal Singh

**Affiliations:** 1 Department of Medicine, Johns Hopkins University School of Medicine, Baltimore, Maryland, United States of America; 2 Department of Epidemiology, The Johns Hopkins University Bloomberg School of Public Health, Baltimore, Maryland, United States of America; 3 Welch Center for Prevention, Epidemiology, and Clinical Research, Johns Hopkins University, Baltimore, Maryland, United States of America; 4 Department of Health Policy and Management, The Johns Hopkins University Bloomberg School of Public Health, Baltimore, Maryland, United States of America; 5 Department of Public Health Sciences, University of Rochester, Rochester, New York, United States of America; US Army Engineer Research and Development Center, UNITED STATES

## Abstract

**Aim:**

To investigate the feasibility and utility of the Analytic Hierarchy Process (AHP) for medication decision-making in type 2 diabetes.

**Methods:**

We conducted an AHP with nine diabetes experts using structured interviews to rank add-on therapies (to metformin) for type 2 diabetes. During the AHP, participants compared treatment alternatives relative to eight outcomes (hemoglobin A1c-lowering and seven potential harms) and the relative importance of the different outcomes. The AHP model and instrument were pre-tested and pilot-tested prior to use. Results were discussed and an evaluation of the AHP was conducted during a group session. We conducted the quantitative analysis using Expert Choice software with the ideal mode to determine the priority of treatment alternatives.

**Results:**

Participants judged exenatide to be the best add-on therapy followed by sitagliptin, sulfonylureas, and then pioglitazone. Maximizing benefit was judged 21% more important than minimizing harm. Minimizing severe hypoglycemia was judged to be the most important harm to avoid. Exenatide was the best overall alternative if the importance of minimizing harms was prioritized completely over maximizing benefits. Participants reported that the AHP improved transparency, consistency, and an understanding of others’ perspectives and agreed that the results reflected the views of the group.

**Conclusions:**

The AHP is feasible and useful to make decisions about diabetes medications. Future studies which incorporate stakeholder preferences should evaluate other decision contexts, objectives, and treatments.

## Introduction

While metformin is the clear first-line medication for the pharmacologic treatment of type 2 diabetes [1], the choice of add-on medications is vast with 11 additional classes available [2, 3]. Beyond this multitude of choices, each medication class, and even a medication within a class, has different benefits and harms; these treatment-related benefits and harms are often unknown at the time of initial Food and Drug Administration (FDA) approval, and they occur at different time points in the course of therapy.

Since views on the importance of treatment-related outcomes vary across patients, providers, regulatory decision-makers, and other stakeholders, both the probabilities of these heterogeneous outcomes and their importance to stakeholders must be considered when making decisions about diabetes medications. The recent combined statement from the American Diabetes Association and the European Association for the Study of Diabetes on patient-centered approaches to hyperglycemia in patients with type 2 diabetes highlights the importance of shared decision-making that considers all aspects of treatment options, including preferences for benefits and harms [2]. Consistent with this shift in thinking in diabetes management, the FDA issued draft guidance in 2013 on the structured assessment of benefit and risk with the goal of facilitating drug regulatory decisions which are explicit and transparent [4].

Given the complexity of medication decision-making in type 2 diabetes, a quantitative method which integrates evidence on treatment-related benefits and harms with preferences regarding trade-offs between benefits and risks should be useful to support decisions. Multicriteria decision analysis (MCDA) refers to a class of model-based methods particularly useful for benefit-risk analysis in the face of multiple objectives (e.g., treatment-related outcomes) [5].

The Analytic Hierarchy Process (AHP) is a commonly-used MCDA method which incorporates benefits and risks explicitly by combining the importance of differences in probabilities of outcomes related to (treatment) alternatives and the weighting of the importance of those outcomes [6–8]. This method results in a transparent decision making process so that groups or individuals using this method can understand and demonstrate the underpinnings of their decisions, in contrast to standard decision making processes in which the importance of the various components of the decision is not explicit [6]. MCDA techniques can be used to structure complex decisions and improve the transparency of the decision making process. The AHP, pioneered by Saaty, is one such approach that is potentially useful in group decision making [7]. It incorporates quantitative and qualitative criteria into the decision process. After structuring the decision model, a series of pairwise comparisons is used to determine the relative importance of the criteria relative to the decision goal, and the importance of treatment options relative to the criteria. These are combined into a numerical score using a weighting process that accounts for direct and indirect comparisons. Measures of consistency are also available. The approach allows decision makers to make transparent judgments based on numerical scores.

Multi-criteria methods have been extensively used to support individual and group decisions in a wide variety of areas, including medical decision making [9–11]. Several multicriteria approaches can be used to solicit input from stakeholders. These include the multi attribute utility analysis, outranking method, The Technique for Order of Preference by Similarity to Ideal Solution (TOPSIS), Measuring Attractiveness by a Categorical Based Evaluation Technique) iMACBETH and several others. Each of these methods has their own strengths and limitations and could have potentially been used for the study. However, we chose the AHP method because it was quite appropriate for the small group setting and represents a readily accessible way to address a complex problem, particularly in a group setting [12]. Secondly, the reporting of consistency ratio allowed us to check the reliability of the study and prevent users from making inconsistent judgments. Thirdly, we considered it because of the flexibility of AHP to combine both qualitative input with quantitative data in an easily understandable manner. Finally, the collective expertise within our group for conducting AHP for similar problems made it a logical choice.

To investigate the feasibility and utility of the AHP for group decision-making for type 2 diabetes management, we conducted an AHP with diabetes clinical, research, and pharmacotherapeutics experts with the goal of determining a ranking of add-on therapies to metformin for patients with type 2 diabetes.

## Materials and Methods

We followed five overlapping steps to design and complete this AHP (Fig A in S2 File) 1) Develop and refine AHP model; 2) Develop and refine AHP instrument; 3) Conduct and analyze AHP; 4) Cognitive interviewing; and 5) Evaluation of AHP. The cognitive interviewing is the topic of a separate publication currently in preparation and is not discussed in this article. Additional details of this study protocol are available in our published protocol [13].

### Study Population

We recruited a group of nine diabetes experts with varying perspectives from clinical (primary care, endocrinology, and pharmacy), research (epidemiology and clinical trials), and operations (pharmacy and therapeutics) disciplines related to diabetes treatment. Participants were recruited following email invitations, and informed consent was provided as recommended by the Institutional Review Board.

### Development and refinement of the AHP model

We used standard AHP methodology to define and refine the decision context, treatment alternatives, and objectives (i.e., treatment-related benefits and harms) for the AHP model [6, 7]. The decision context consisted of the specific decision goal, the relevant population and the decision-makers, and we limited the options to pharmacologic treatment alternatives. We constructed the AHP model as a hierarchy with the decision goal at the top and treatment alternatives at the bottom [7]. The level of the hierarchy below the decision goal comprised the general objectives with more specific objectives placed below the general objectives [7]. We aimed for seven or fewer objectives on a given level to reduce the number of comparisons and thus reduce respondent burden and improve consistency. We framed objectives positively (e.g., “maximize benefit” and “minimize harms”). At each level of the hierarchy, objectives are compared to one another in a pairwise fashion to determine their importance weights. Treatment alternatives are then compared to one another in a pairwise fashion taking into consideration their relative ability to fulfill the criteria; these results are termed “judgments”.

We used the most current FDA label information (www.fda.gov) for each treatment alternative to obtain data on the objectives and supplemented this with results from a Comparative Effectiveness Review of diabetes medications developed under contract from the Agency for Healthcare Quality and Research (Table A in S1 File) [14]. We evaluated multiple visual representations of the treatment-specific quantitative evidence based on prior work [15] and selected bar charts for use during the actual AHP sessions. We presented data on objectives with either metformin or placebo/usual care as the reference depending on the availability of data.

### Development and refinement of the AHP instrument

The AHP hierarchy was entered into Expert Choice, a widely-used software package, which has a web-based platform and can perform analyses in real time [16]. This process translated the hierarchy into a series of questions asking participants to judge the relative weights of alternatives and objectives in relation to the objectives just above them in the hierarchy [6, 7, 17]. The wording and presentation of questions was customized for this application, including the presentation of information for the participants that described the decision context and the hierarchical model, and a brief explanation of the AHP method.

We validated and refined the decision context, model content, and hierarchy through in-person group sessions with our panel of experts on two occasions (pilot sessions lasted 60–90 minutes). During the pilot, expert participants made comparisons among the alternatives or objectives at each level of the hierarchy by entering direct numeric weights (numbers between 0 and 1) which were then transformed by the software to the usual AHP scale using the standard eigenvector procedure [18, 19]. We also distributed a list of three open-ended questions about the model to participants during these sessions (Fig B in S2 File) and incorporated expert feedback into a revised version of the decision context and AHP model.

In response to initial expert feedback, we simplified the AHP model (limited the number of objectives to those on which data were available and included only medications considered second-line in the recent statement on management of hyperglycemia [2]; (Fig C in S2 File) and specified a patient population, the type of decision being made (e.g., regulatory or for an individual patient), and the relevant step in the sequence of therapy (first-line versus add-on) (Fig D in S2 File). Although our preliminary model included all medications and outcomes, all medication options and outcomes could not be included in the final AHP model. Pragmatic considerations, input from experts and the specific decision context dictated the final choice of medications and outcomes. As an example, rosiglitazone was excluded because of lack of use in the United States. Lactic acidosis, a well-known complication, was excluded because it was too rare and had an unknown incidence.

The simplified task had pair-wise rather than direct comparisons and fewer questions, and the wording was changed to be more intuitive. We also added a detailed welcome page that outlined the decision context, showed the full hierarchy, and explained the task in more detail, and presented the data for treatment-related outcomes (evidence matrix). In order to make the ratio scale more salient, we provided participants with the option of making their pairwise comparisons using numeric or graphical data entry (Fig E in S2 File).

### Conduct and analysis of the AHP

For the conduct of the final AHP, we sent participants a web link to the AHP instrument with instructions to complete it prior to a consensus group session. Relative weights from pairwise comparisons of objectives were obtained by calculation of the right principal eigenvector of the relevant matrix (e.g., matrix of the pairwise comparisons between objectives at one level of the hierarchy). Expert Choice uses the matrix multiplication method, considered to be accurate, for this calculation [6]. We used the ideal synthesis mode which is designed to identify the single best alternative or most important criterion. An advantage of this mode is that relative ranks are preserved in the case of the addition or removal of an 'irrelevant' alternative [20]. Similar calculations were performed to obtain weights for treatment-specific evidence on the objectives.

The priority of a given treatment alternative with respect to meeting an objective at the next level up in the hierarchy was obtained by summing the products of the weight for the alternative with respect to the objective and each objective weight at the level below in the hierarchy. Priorities for alternatives were compared using ratios with relative differences of 1.1 considered significant according to standard AHP criteria [7]. A ratio of 1.1 between two alternatives implies a 10% multiplicative difference with respect to how the alternatives meet a given objective at the next level above in the hierarchy. Priorities for objectives were calculated and interpreted similarly. Group priorities were calculated using the geometric mean of the individual experts’ priorities.

We performed sensitivity analyses to understand the impact of particular objectives and weights on our results: 1) Increasing the priority of maximizing benefits to 100%; 2) Increasing the priority of minimizing harms to 100%; and 3) Conducting the analysis in the distributive mode. In contrast to the ideal mode, the distributive mode produces results that evaluate alternatives or criteria proportionately [20]. This characteristic makes the distributive mode more suitable for identifying relative priorities among criteria or alternatives but also makes results dependent on the composition of the set of alternatives or criteria being compared.

We anticipated heterogeneity in weights of objectives and alternatives across participants and evaluated standard deviations (indicating the extent of agreement or disagreement) for weights.

We used the consistency index to evaluate consistency, or transitivity, of weights [17]. A perfectly consistent set of comparisons has a consistency index of 0. Based on generally accepted convention, we considered weights to be inconsistent if consistency index values exceeded 0.15 [17].

#### Evaluation of the AHP

We invited participants to a group session to review and discuss results. Respondents were also asked to complete an evaluation form to provide feedback on the process. Feedback was analyzed to tally responses and identify themes.

### Ethics

Participants were recruited for the group sessions by a recruitment email sent out by the study team to a group of experts. The participants provided oral consent for the study using an oral consent script. Written consent was not sought to ensure the anonymity of participants. Their consent was noted separately without identifiers. The overall study and recruitment email, and consent procedure were approved by the Institutional Review Board of Johns Hopkins University School of Medicine.

## Results

Group priority scores for all objectives are shown in Table 1. Overall, maximizing benefits was judged to be 21% more important than minimizing harm. Reducing HbA1c was judged to be 3.71 and 1.80 times more important than minimizing non-serious and serious harms, respectively, and minimizing serious harms was more than twice as important as minimizing non-serious harms (Table A and Table B in S1 File). Relative differences between objectives at the lowest level of the hierarchy are shown in Table 2. Most notably, all objectives were judged to be significantly more important than minimizing fracture risk; minimizing severe hypoglycemia was judged to be significantly more important than all other objectives; and the largest relative difference in importance was for minimizing severe hypoglycemia versus minimizing fracture risk (relative difference, 5.45).

**Table 1:** Global priority scores of objectives.

Objective	Global Priority Score, %
Maximizing benefits	54.83
Reduce HbA1c	54.83
Minimizing harms	45.17
Minimizing non-serious harms	14.79
Risk of fracture	2.57
Weight gain	7.65
GI symptoms	4.57
Minimizing serious harms	30.38
Severe hypoglycemia	14.01
CHF risk	7.96
Acute pancreatitis	4.46
Risk of bladder cancer	3.95

**Table 2:** Relative differences in importance between objectives at lowest level of hierarchy.*

	Fracture	Weight gain	GI symptoms	Severe hypoglycemia	CHF	Acute pancreatitis	Bladder cancer
**Fracture**	1.0						
**Weight gain**	**2.98**	1.0				**1.72**	**1.94**
**GI symptoms**	**1.78**	**1.67**	1.0			1.02	**1.16**
**Severe hypoglycemia**	**5.45**	**1.83**	**3.07**	1.0	**1.76**	**3.14**	**3.55**
**CHF**	**3.10**	1.04	**1.74**		1.0	**1.78**	**2.02**
**Acute pancreatitis**	**1.74**					1.0	**1.13**
**Bladder cancer**	**1.54**						1.0

*Relative difference is calculated as ratio of global priority scores shown in Table 1 (e.g., relative difference for minimizing severe hypoglycemia versus minimizing fracture risk is 14.0/2.57 = 5.45)

**Fig 1:**
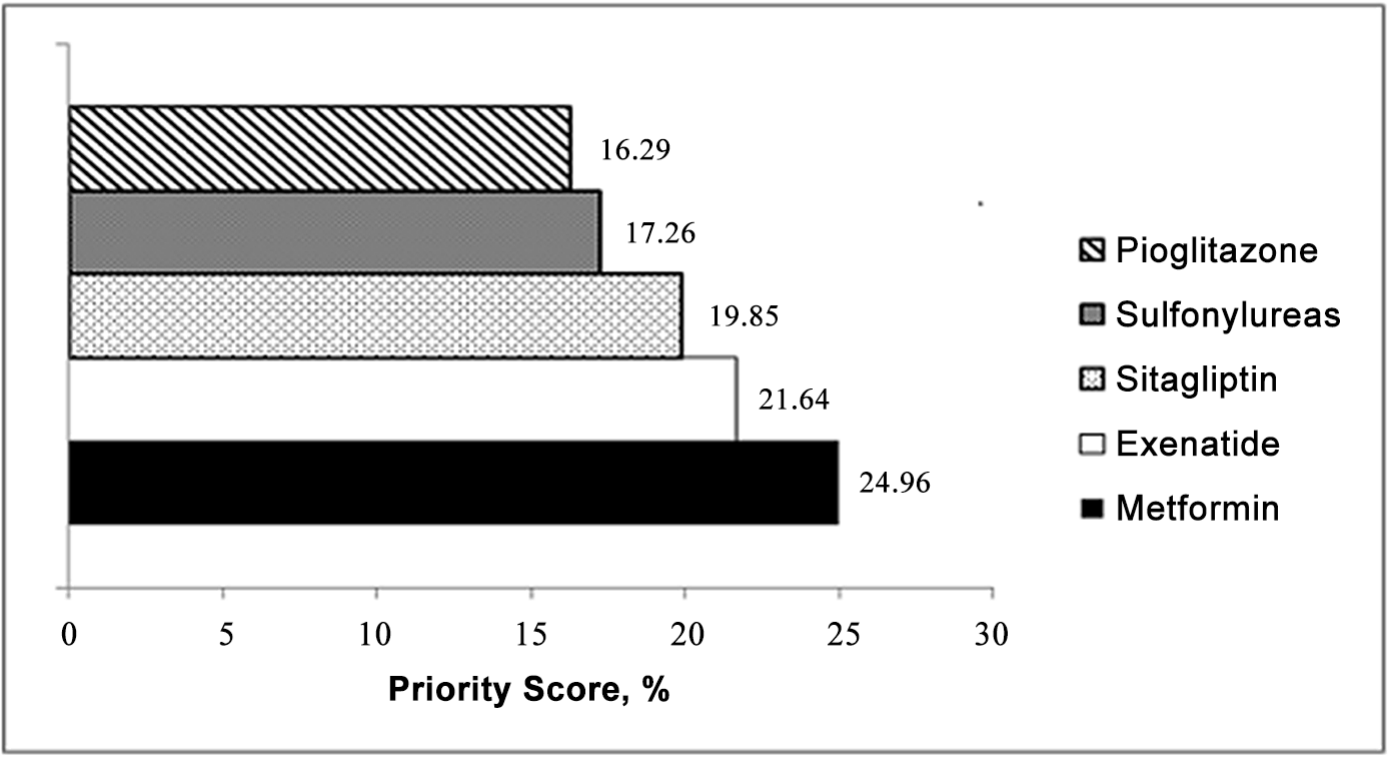
Overall priority scores for treatment alternatives.

Priority scores for the alternatives showed that exenatide was the preferred add-on alternative followed by sitagliptin, sulfonylureas, and then pioglitazone (Fig 1). Relative differences for metformin versus all alternatives were greater than 1.1 so were considered significant (Table 3). Exenatide and sitagliptin were also judged to perform significantly better than sulfonylureas and pioglitazone (Table 3).

**Table 3:** Overall relative differences between treatment alternatives.*

	Metformin	Exenatide	Sitagliptin	Sulfonylureas	Pioglitazone
Metformin	1.00	**1.15**	**1.26**	**1.45**	**1.53**
Exenatide		1.00	1.09	**1.25**	**1.33**
Sitagliptin			1.00	1.15	1.22
Sulfonylureas				1.00	1.06
Pioglitazone					1.00

*Relative difference is calculated as ratio of global priority scores shown in Table 1 (e.g., relative difference for minimizing severe hypoglycemia versus minimizing fracture risk is 14.0/2.57 = 5.45)

Priorities for each treatment meeting individual objectives are shown in Table C in S1 File, and relative differences between alternatives in Table D, Table E, Table F, Table, G, Table H, Table I, Table J and Table K in S1 File. Metformin was preferred significantly over all other treatments for reducing HbA1c (range in relative differences, 1.48 to 1.70), and exenatide was preferred significantly more than pioglitazone for HbA1c reduction (relative difference, 1.15). All treatments were preferred significantly more than pioglitazone for minimizing fracture risk; CHF; and risk of bladder cancer (relative differences >1.1). Exenatide, metformin, and sitagliptin were preferred compared to sulfonylureas and pioglitazone for minimizing severe hypoglycemia (relative differences >1.1). Exenatide was considered significantly better than all other treatments for minimizing weight gain, followed by metformin (relative differences >1.1). Exenatide and sitagliptin were also judged to perform significantly better for minimizing CHF compared to sulfonylureas. Pioglitazone, sulfonylureas, and sitagliptin outperformed metformin and exenatide for minimizing GI symptoms. Relative differences between treatments were not different for minimizing acute pancreatitis.

The sensitivity analysis prioritizing maximizing benefits (weight: 100%) over minimizing harms (weight: 0%) did not alter the ranking of the treatment alternatives (Fig F in S2 File). However, prioritizing minimizing harms (weight: 100%) over maximizing benefits (weight: 0%) changed the ranking so that exenatide was preferred followed by sitagliptin, metformin, sulfonylureas, and then pioglitazone (Fig G in S2 File). Conducting the analysis using the distributive mode did not change the ranking of medications.

We observed the largest variation in the experts’ priorities for minimizing weight gain versus minimizing non-serious harms (SD, 24.5%), the performance of exenatide in minimizing weight gain (SD, 20.9%), and the reduction of HbA1c with metformin (SD, 20.7%). We observed the smallest variation for the performance of sulfonylureas and metformin at minimizing risk of bladder cancer (SD, 0.48% and 0.51%, respectively). Consistency ratios for most participants and judgments were less than <0.1 indicating low levels of inconsistency.

All respondents noted that the process was beneficial for building consensus through improving transparency, consistency, and an understanding of others’ perspectives. Respondents agreed that the ranking and weighting of medications and objectives reflected the views of the group. Almost all respondents agreed that this method could be adapted to take into account new information on risks and benefits. Views on the ease of interpreting the AHP results were more heterogeneous with ratings ranging between the ease of interpretation being “very clear” to “not straightforward at all.”

## Discussion

We demonstrate that the AHP can be used to facilitate group decision-making about medications for type 2 diabetes and that the underpinnings of the decision are transparent and clear to participants. For example, metformin was preferred by participants given the treatment-related outcomes included in the model, and the rank order derived from the judgments was sensitive to whether minimizing risks was prioritized completely over maximizing benefit. Although participants did have reservations about the process, they certainly noted benefits to using the AHP to facilitate decision making.

The AHP has not been used previously to make decisions about diabetes medications to our knowledge, but it has been used in a variety of other applications, in health care, regulatory science, and other decision-making contexts [8, 21].

Decision-making to select medications to add on to metformin for type 2 diabetes is complex because of the numerous treatment alternatives; their varied benefits and harms; the timing of their benefits and harms; and the varied stakeholder preferences for those benefits and harms. For example, the hemoglobin A1c-lowering effect is estimated to be 1–1.5% for metformin, sulfonylureas, TZD (Thiazolidinediones), and GLP-1 (Glucagon Like Peptide-1) agonists but only 0.5–1% for DPP-4 (Dipeptidyl peptidase-4) inhibitors [2]; thus, importance of HbA1c-lowering relative to medication risks must be considered when making treatment decisions. Patients with limited life expectancy and their providers may be less concerned with HbA1c-lowering and more concerned about medication side effects. Also, the evidence on the risk of bladder cancer with pioglitazone began to emerge approximately six years after pioglitazone was approved by the FDA [22–25]. Although bladder cancer is likely to be rare, it was clear from our results that participants did attach some importance to this new outcome, and the AHP is flexible in that new treatment-related outcomes of this nature can be incorporated to facilitate decisions. Theoretical outcomes can also be incorporated in the absence of clear evidence.

A major strength of our study is the iterative process used to develop the AHP model and instrument. While participants were clinical or research experts in diabetes, they did not have experience with the AHP, and with feedback and refinement, we conducted an AHP with results that were reasonable to participants. Our protocol can be easily translated to other decision contexts.

While the results of this AHP only apply to the particular decision context we specified, they are not meant to be generalizable. The goal of this study was not to declare a ranking of the treatment alternatives but to demonstrate how the AHP can be used to facilitate decisions. Furthermore, the AHP does not replace additional judgment; it simply provides a framework for explicitly considering the relative importance and effects of treatment-related outcomes and a clear starting point for making decisions.

Our model for this AHP certainly excludes important outcomes such as macro- and microvascular complications, quality of life, and cost as well as many treatment alternatives. We removed many of these from our model in response to piloting with diabetes experts and decided to rely on HbA1c as the main benefit and exclude consideration of cost to be consistent with the FDA convention to making decisions about diabetes medications. We were also limited by the lack of data on many outcomes such as quality of life, and sought to limit the number of objectives to maintain decisional capacity and minimize respondent burden. Additional objectives and treatment alternatives could be added to the model.

Although we chose AHP, there are limitations of the AHP approach. Firstly, AHP is a time-intensive process. Secondly, structuring of the problem such that criteria with large number of sub criteria receive more weights needs to be considered. Thirdly, some argue that the ratio scale also does not allow a zero when the verbal ratings are classified into 1–9 integer scale. Fourthly, the preferred alternative may change because of the introduction or deletion of an alternative when the distributive mode of synthesis is being used. This has been shown to be a potential problem with all additive multicriteria models. It is less of a problem when the ideal mode of synthesis is used and can also be viewed as an appropriate outcome based on the changes in the problem being addressed and therefore a strength, rather than a limitation, of the method. Finally another limitation of the AHP is that weights for objectives and treatment alternatives are assumed to be independent, but this may not be the case. For example, if a participant believes that exenatide lowers weight dramatically and also believes this is very important, then this may affect the way that he/she makes other comparisons as well. To reduce this chance, we asked participants to make comparisons regarding treatment-related probabilities prior to weighting the actual outcomes. Although we demonstrated lack of inconsistency in judgments for the majority of participants and face validity, the small of number of participants limits generalizability. This did not allow us to detect differences in priorities among subgroups. This should be done with larger samples. We used geometric mean of the individual expert’s priorities to estimate group priorities.

While other methods are also available, they have their own strengths and limitations. The multiattribute utility analysis could have been a potential option. However the assumptions, such as the decision maker’s ability to make reasonable judgments about swing judgments, and the appropriateness of using priority weights based on ordinal rankings have not been tested. Since the comparisons of one method to another are few and sensitive to the decision context, future studies should conduct such head to head evaluations.

The particular software-Expert Choice used for this estimation did not report on the variance. Other limitations included that Expert Choice [16], the software we used to conduct this AHP, has some limitations in flexibility of editing the user interface to promote usability for those unfamiliar with the process. Also, we primarily used FDA label information for this study, but participants sometimes disagreed with these results. Finally, the visual representation that we used for presenting treatment-related outcome data may not have been ideal for all participants.

Future studies should evaluate the feasibility of the AHP for other decision contexts in diabetes. While we used the AHP to facilitate a group decision regarding a medication formulary, the decision goal could certainly be modified to address other group decisions such as ones at the regulatory level, and patients and their providers could also use this process to make individual clinical decisions.

## Conclusions

In summary, we demonstrate that the AHP can be used to facilitate a group decision about add-on therapy to metformin for patients with type 2 diabetes. This process allows us to understand the importance decision makers assign to the different elements of the decision including the importance of differences in probabilities of the treatment-related outcomes, the relative importance of treatment-related outcomes, and the sensitivity of the medication rankings to manipulation of these. The AHP thus presents a feasible tool for improving patient-centered care in type 2 diabetes by explicitly incorporating information about all aspects of a decision, including preference for treatment-related outcomes.

We have demonstrated the reliability and face validity of a small group decision making approach such as AHP in making informed and transparent choices about treatment. While AHP has been conducted in various other health care settings, the approach outlined above can be used to improve the transparency of regulatory decisions around benefits and risks of products.

## Supporting Information

S1 File Supporting tables. Table A in S1 File. Data on objectives for treatment alternatives. Table B in S1 File. Relative differences between objectives at third level of hierarchy*. Table C in S1 File. Treatment priorities by objectives. Table D in S1 File. Relative differences between alternatives for maximizing reduction of HbA1c. Table E in S1 File. Relative differences between alternatives for minimizing risk of fracture. Table F in S1 File. Relative differences between alternatives for minimizing weight gain. Table G in S1 File. Relative differences between alternatives for minimizing GI symptoms. Table H in S1 File. Relative differences between alternatives for minimizing risk of severe hypoglycemia. Table I in S1 File. Relative differences between alternatives for minimizing risk of CHF. Table J in S1 File. Relative differences between alternatives for minimizing risk of acute pancreatitis. Table K in S1 File. Relative differences between treatment alternatives for minimizing risk of bladder cancer.
(DOC)

S2 File Supporting Figures. Fig A in S2 File. Process used for development and conduct of the Analytic Hierarchy Process. Fig B in S2 File Group Session Feedback Questions. Fig C in S2 File. Final Analytic Hierarchy Process Model. Fig D in S2 File. Analytic Hierarchy Process Case Scenario. Fig E in S2 File. Example of interface used to obtain user input on relative importance of clinical differences in HbA1c-lowering for treatment alternatives. Fig F in S2 File. Overall global priorities when maximizing benefit completely over minimizing harm. Fig G in S2 File. Overall global priorities when minimizing harms completely over maximizing benefits.
(DOCX)
