# Supplementary material for: Use of the Analytic Hierarchy Process for Medication Decision-Making in Type 2 Diabetes
Source: PLoS One. 2015 May 22;10(5):e0126625. doi: 10.1371/journal.pone.0126625 (PMC4441461; doi:10.1371/journal.pone.0126625)
Supplement: S1 File — Table A in S1 File. Data on objectives for treatment alternatives. Table B in S1 File. Relative differences between objectives at third level of hierarchy*. Table C in S1 File. Treatment priorities by objectives. Table D in S1 File. Relative differences between alternatives for maximizing reduction of HbA1c. Table E in S1 File. Relative differences between alternatives for minimizing risk of fracture. Table F in S1 File. Relative differences between alternatives for minimizing weight gain. Table G in S1 File. Relative differences between alternatives for minimizing GI symptoms. Table H in S1 File. Relative differences between alternatives for minimizing risk of severe hypoglycemia. Table I in S1 File. Relative differences between alternatives for minimizing risk of CHF. Table J in S1 File. Relative differences between alternatives for minimizing risk of acute pancreatitis. Table K in S1 File. Relative differences between treatment alternatives for minimizing risk of bladder cancer. (DOC) [file pone.0126625.s001.doc]

**Table A in S1 File. Data on objectives for treatment alternatives**

|  | **Metformin vs. placebo** | **Sulfonylureas vs. metformin^a^** | **Pioglitazone vs. metformin^b^** | **Exenatide vs. metformin** | **Sitagliptin vs. metformin** |
| --- | --- | --- | --- | --- | --- |
| Mean HbA1c change | -1.80% | -0.07% | -0.50% | -0.90% | -0.80% |
| Mean weight change | +0.5 kg | +2.7 kg | +2.6 kg^a^ | -2.4 kg | 0 kg |
| Fracture | 0 | 0 | 2-3 in 100 (women) | 0 | n/a |
| Incident GI symptoms | 40 in 100^c^ | 27 in 100 fewer^c^ | 0 | 26 in 100^d^ | 2-3 in 100^e^ |
| Severe Hypoglycemia | 0 | 11 in 100^f^ | 8-9 in 100 | 0 | 8 in 1000 |
| CHF | 0 | 8 in 1000^g^ | 1-2 in 100 | 0 | 0 |
| Acute pancreatitis | 0 | 0 | 0 | 262 cases, denominator | 0.1 cases / 100 pt-yrs^e^ |
| Bladder cancer | 0 | 0 | 3 in 1000 | 0 | 0 |
| ^a^ Bennett, W. L., et al. (2011). "Comparative effectiveness and safety of medications for type 2 diabetes: an update including new drugs and 2-drug combinations." Ann Intern Med **154**(9): 602-613. | | | | | |
| ^b^ Results from randomized trial in which participants continued to take prior antidiabetic medications including metformin (Dormandy, J. A., et al. (2005). "Secondary prevention of macrovascular events in patients with type 2 diabetes in the PROactive Study (PROspective pioglitAzone Clinical Trial in macroVascular Events): A randomised controlled trial." Lancet **366**(9493): 1279-1289). | | | | | |
| ^c^ Diarrhea |  |  |  |  |  |
| ^d^ Incidence of nausea when exenatide is added to metformin and/or SU vs. (metformin and/or SU) in 3 RCTs | | | | |  |
| ^e^ Versus placebo or other antidiabetic medication therapy | | |  |  |  |
| ^f^ Mild or moderate hypoglycemia | |  |  |  |  |
| ^g^ Absolute rate in SU arms from trials of SU vs. TZD (Bennett, W. L., et al. (2011). "Comparative effectiveness and safety of medications for type 2 diabetes: an update including new drugs and 2-drug combinations." Ann Intern Med **154**(9): 602-613.) | | | | | |

**Table B in S1 File**. Relative differences between objectives at third level of hierarchy[[1]](#endnote-2)

|  | **Reduce HbA1c** | **Minimize serious harms** | **Minimize non-serious harms** |
| --- | --- | --- | --- |
| **Reduce HbA1c** | 1.0 | **1.80** | **3.71** |
| **Minimize serious harms** |  | 1.0 | **2.05** |
| **Minimize non-serious harms** |  |  | 1.0 |

**Table C in S1 File**. Treatment priorities by objectives

| **Objective** | **Treatment** | **Treatment Priority** |
| --- | --- | --- |
| Maximize benefits: Reduce HbA1c |  |  |
|  | Metformin 100.00 % | 100.00 % |
|  | Exenatide 67.90 % | 67.90 % |
|  | Sitagliptin 63.78 % | 63.78 % |
|  | Sulfonylureas 62.16 % | 62.16 % |
|  | Pioglitazone 59.13 % | 59.13 % |
| Minimize harms |  |  |
| Minimize non-serious harms |  |  |
| Minimize risk of fracture |  |  |
|  | Metformin 100.00 % | 100.00 % |
|  | Sitagliptin 63.78 % | 98.48 % |
|  | Sulfonylureas 62.16 % | 96.12 % |
|  | Exenatide 67.90 % | 93.66 % |
|  | Pioglitazone 59.13 % | 39.29 % |
| Minimize weight gain |  |  |
|  | Exenatide 67.90 % | 100.00 % |
|  | Metformin 100.00 % | 43.18 % |
|  | Sitagliptin 63.78 % | 39.35 % |
|  | Sulfonylureas 62.16 % | 19.24 % |
|  | Pioglitazone 59.13 % | 19.06 % |
| Minimize GI symptoms |  |  |
|  | Pioglitazone 59.13 % | 100.00 % |
|  | Sitagliptin 63.78 % | 91.16 % |
|  | Sulfonylureas 62.16 % | 83.35 % |
|  | Metformin 100.00 % | 44.85 % |
|  | Exenatide 67.90 % | 43.39 % |
| Minimize harms: |  |  |
| Minimize serious harms |  |  |
| Minimize risk of severe hypoglycemia |  |  |
|  | Exenatide 67.90 % | 100.00 % |
|  | Metformin 100.00 % | 91.18 % |
|  | Sitagliptin 63.78 % | 86. 79 % |
|  | Pioglitazone 59.13 % | 64.43 % |
|  | Sulfonylureas 62.16 % | 43.94 % |
| Minimize risk of CHF |  |  |
|  | Exenatide 67.90 % | 100.00 % |
|  | Sitagliptin 63.78 % | 98.82 % |
|  | Metformin 100.00 % | 95.75 % |
|  | Sulfonylureas 62.16 % | 87.45 % |
|  | Pioglitazone 59.13 % | 44.12 % |
| Minimize risk of acute pancreatitis |  |  |
|  | Metformin 100.00 % | 100.00 % |
|  | Sulfonylureas 62.16 % | 99.73 % |
|  | Pioglitazone 59.13 % | 98.72 % |
|  | Exenatide 67.90 % | 93.85 % |
|  | Sitagliptin 63.78 % | 92.51 % |
| Minimize risk of bladder cancer |  |  |
|  | Sitagliptin 63.78 % | 100.00 % |
|  | Metformin 100.00 % | 99.60 % |
|  | Exenatide 67.90 % | 99.52 % |
|  | Sulfonylureas 62.16 % | 99.18 % |
|  | Pioglitazone 59.13 % | 84.91 % |

**Table D in S1 File.** Relative differences between alternatives for maximizing reduction of HbA1c[[2]](#footnote-2)

|  | Metformin | Exenatide | Sitagliptin | Sulfonylureas | Pioglitazone |
| --- | --- | --- | --- | --- | --- |
| Metformin | 1.00 | **1.48** | **1.58** | **1.63** | **1.70** |
| Exenatide |  | 1.00 | 1.06 | 1.09 | **1.15** |
| Sitagliptin |  |  | 1.00 | 1.03 | 1.08 |
| Sulfonylureas |  |  |  | 1.00 | 1.04 |
| Pioglitazone |  |  |  |  | 1.00 |

**Table E in S1 File.** Relative differences between alternatives for minimizing risk of fracture[[3]](#footnote-3)

|  | Metformin | Exenatide | Sitagliptin | Sulfonylureas | Pioglitazone |
| --- | --- | --- | --- | --- | --- |
| Metformin | 1.00 | 1.07 | 1.01 | 1.04 | **2.54** |
| Exenatide |  | 1.00 | 0.95 | 0.97 | **2.38** |
| Sitagliptin |  |  | 1.00 | 1.02 | **2.51** |
| Sulfonylureas |  |  |  | 1.00 | **2.45** |
| Pioglitazone |  |  |  |  | 1.00 |

**Table F in S1 File.** Relative differences between alternatives for minimizing weight gain[[4]](#footnote-4)

|  | Metformin | Exenatide | Sitagliptin | Sulfonylureas | Pioglitazone |
| --- | --- | --- | --- | --- | --- |
| Metformin | 1.00 | **0.43** | 1.09 | **2.24** | **2.26** |
| Exenatide |  | 1.00 | **2.54** | **5.19** | **5.24** |
| Sitagliptin |  |  | 1.00 | **2.04** | **2.06** |
| Sulfonylureas |  |  |  | 1.00 | 1.01 |
| Pioglitazone |  |  |  |  | 1.00 |

**Table G in S1 File.** Relative differences between alternatives for minimizing GI symptoms[[5]](#footnote-5)

|  | Metformin | Exenatide | Sitagliptin | Sulfonylureas | Pioglitazone |
| --- | --- | --- | --- | --- | --- |
| Metformin | 1.00 | 1.03 | **0.49** | **0.54** | **0.45** |
| Exenatide |  | 1.00 | **0.47** | **0.52** | **0.43** |
| Sitagliptin |  |  | 1.00 | 1.09 | 0.91 |
| Sulfonylureas |  |  |  | 1.00 | 0.83 |
| Pioglitazone |  |  |  |  | 1.00 |

**Table H in S1 File.** Relative differences between alternatives for minimizing risk of severe hypoglycemia[[6]](#footnote-6)

|  | Metformin | Exenatide | Sitagliptin | Sulfonylureas | Pioglitazone |
| --- | --- | --- | --- | --- | --- |
| Metformin | 1.00 | 0.91 | 1.05 | **2.07** | **1.41** |
| Exenatide |  | 1.00 | **1.15** | **2.27** | **1.55** |
| Sitagliptin |  |  | 1.00 | **1.97** | **1.35** |
| Sulfonylureas |  |  |  | 1.00 | **0.68** |
| Pioglitazone |  |  |  |  | 1.00 |

**Table I in S1 File.** Relative differences between alternatives for minimizing risk of CHF[[7]](#footnote-7)

|  | Metformin | Exenatide | Sitagliptin | Sulfonylureas | Pioglitazone |
| --- | --- | --- | --- | --- | --- |
| Metformin | 1.00 | 0.95 | 0.97 | 1.09 | **2.17** |
| Exenatide |  | 1.00 | 1.01 | **1.14** | **2.27** |
| Sitagliptin |  |  | 1.00 | **1.13** | **2.24** |
| Sulfonylureas |  |  |  | 1.00 | **1.98** |
| Pioglitazone |  |  |  |  | 1.00 |

**Table J in S1 File.** Relative differences between alternatives for minimizing risk of acute pancreatitis[[8]](#footnote-8)

|  | Metformin | Exenatide | Sitagliptin | Sulfonylureas | Pioglitazone |
| --- | --- | --- | --- | --- | --- |
| Metformin | 1.00 | 1.06 | 1.08 | 1.00 | 1.01 |
| Exenatide |  | 1.00 | 1.01 | 0.94 | 0.95 |
| Sitagliptin |  |  | 1.00 | 0.93 | 0.94 |
| Sulfonylureas |  |  |  | 1.00 | 1.01 |
| Pioglitazone |  |  |  |  | 1.00 |

**Table K in S1 File.** Relative differences between treatment alternatives for minimizing risk of bladder cancer[[9]](#footnote-9)

|  | Metformin | Exenatide | Sitagliptin | Sulfonylureas | Pioglitazone |
| --- | --- | --- | --- | --- | --- |
| Metformin | 1.00 | 1.00 | 0.99 | 1.00 | **1.17** |
| Exenatide |  | 1.00 | 0.99 | 1.00 | **1.17** |
| Sitagliptin |  |  | 1.00 | 1.01 | **1.18** |
| Sulfonylureas |  |  |  | 1.00 | **1.17** |
| Pioglitazone |  |  |  |  | 1.00 |

1. Relative difference is calculated as ratio of global priority scores from Table 1 (e.g., relative difference for reducing HbA1c versus minimizing serious harms is 54.83/30.38 = 1.80) [↑](#endnote-ref-2)
2. Relative difference is calculated as ratio of global priority scores from Supplemental Table S3 (e.g., relative difference for exenatide versus pioglitazone for maximizing reduction of HbA1c is 67.90/59.13 = 1.15) [↑](#footnote-ref-2)
3. Relative difference is calculated as ratio of global priority scores from Supplemental Table S3 (e.g., relative difference for exenatide versus pioglitazone for minimizing risk of fracture is 93.66/39.29 = 2.38) [↑](#footnote-ref-3)
4. Relative difference is calculated as ratio of global priority scores from Supplemental Table S3 (e.g., relative difference for sulfonylureas versus pioglitazone for minimizing weight gain is 19.24/19.06= 1.01) [↑](#footnote-ref-4)
5. Relative difference is calculated as ratio of global priority scores from Supplemental Table S3 (e.g., relative difference for exenatide versus pioglitazone for minimizing GI symptoms is 43.39/100.00= 0.43) [↑](#footnote-ref-5)
6. Relative difference is calculated as ratio of global priority scores from Supplemental Table S3 (e.g., relative difference for exenatide versus sulfonylureas for minimizing risk of severe hypoglycemia is 100.00/43.94= 2.27) [↑](#footnote-ref-6)
7. Relative difference is calculated as ratio of global priority scores from Supplemental Table S3 (e.g., relative difference for sulfonylureas versus pioglitazone for minimizing risk of CHF is 87.45/44.12= 1.98) [↑](#footnote-ref-7)
8. Relative difference is calculated as ratio of global priority scores from Supplemental Table S3 (e.g., relative difference for exenatide versus sulfonylureas for minimizing risk of acute pancreatitis is 93.85/99.73= 0.94) [↑](#footnote-ref-8)
9. Relative difference is calculated as ratio of global priority scores from Supplemental Table S3 (e.g., relative difference for sitagliptin versus pioglitazone for minimizing risk of bladder cancer is 100.00/84.91= 1.18) [↑](#footnote-ref-9)
