# Supplementary material for: Use of the Analytic Hierarchy Process for Medication Decision-Making in Type 2 Diabetes
Source: PLoS One. 2015 May 22;10(5):e0126625. doi: 10.1371/journal.pone.0126625 (PMC4441461; doi:10.1371/journal.pone.0126625)
Supplement: S2 File — Fig A in S2 File. Process used for development and conduct of the Analytic Hierarchy Process. Fig B in S2 File Group Session Feedback Questions. Fig C in S2 File. Final Analytic Hierarchy Process Model. Fig D in S2 File. Analytic Hierarchy Process Case Scenario. Fig E in S2 File. Example of interface used to obtain user input on relative importance of clinical differences in HbA1c-lowering for treatment alternatives. Fig F in S2 File. Overall global priorities when maximizing benefit completely over minimizing harm. Fig G in S2 File. Overall global priorities when minimizing harms completely over maximizing benefits. (DOCX) [file pone.0126625.s002.docx]

**Figure A in S2 File**. Process used for development and conduct of the Analytic Hierarchy Process

Develop hierarchy

Feedback on hierarchy

Revise hierarchy

Develop AHP instrument

AHP pilot

Develop evidence matrix

Refine AHP instrument

AHP data collection

Develop cognitive debrief protocol

Cognitive debriefing

AHP evaluation

Analysis

**Figure B in S2 File**. Group Session Feedback Questions

**Figure C in S2 File**. Final Analytic Hierarchy Process Model

**Figure D in S2 File**. Analytic Hierarchy Process Case Scenario

**Figure E in S2 File.** Example of interface used to obtain user input on relative importance of clinical differences in HbA1c-lowering for treatment alternatives


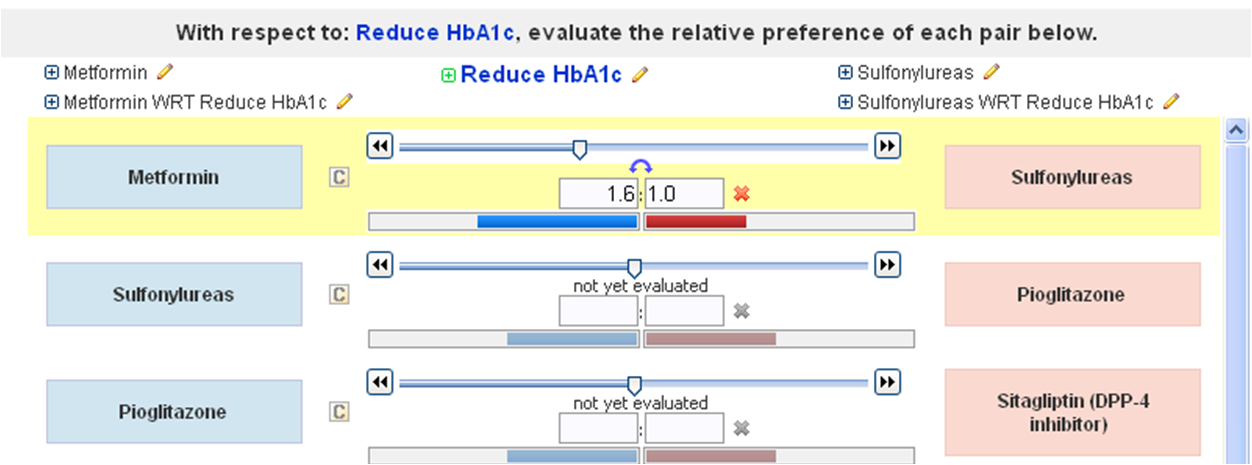


**Figure F in S2 File**. Overall global priorities when maximizing benefit completely over minimizing harm

**Figure G in S2 File.** Overall global priorities when minimizing harms completely over maximizing benefits
